# Induction of the Inflammasome Pathway by Tyrosine Kinase Inhibitors Provides an Actionable Therapeutic Target for Hepatocellular Carcinoma

**DOI:** 10.3390/cancers16081491

**Published:** 2024-04-13

**Authors:** Anna Tutusaus, Marco Sanduzzi-Zamparelli, Loreto Boix, Patricia Rider, Silvia Subías, Pablo García de Frutos, Anna Colell, Montserrat Marí, María Reig, Albert Morales

**Affiliations:** 1 Department of Cell Death and Proliferation, IIBB-CSIC, Institut d’Investigacions Biomèdiques August Pi i Sunyer (IDIBAPS), 08036 Barcelona, Spain; anna.tutusaus@iibb.csic.es (A.T.); patricia.rider@iibb.csic.es (P.R.); pablo.garcia@iibb.csic.es (P.G.d.F.); anna.colell@iibb.csic.es (A.C.); montserrat.mari@iibb.csic.es (M.M.); 2 Barcelona Clinic Liver Cancer (BCLC) Group, Institut d’Investigacions Biomèdiques August Pi i Sunyer (IDIBAPS), 08036 Barcelona, Spain; msanduzzi@clinic.cat (M.S.-Z.); lboix@clinic.cat (L.B.); 3 Liver Unit, Hospital Clinic de Barcelona, Institut d’Investigacions Biomèdiques August Pi i Sunyer (IDIBAPS), 08036 Barcelona, Spain; 4 Centro de Investigación Biomédica en Red de Enfermedades Hepáticas y Digestivas, CIBEREHD, ISCIII, 28029 Madrid, Spain; 5 Departament de Medicina, Facultat de Medicina, Universitat de Barcelona, 08036 Barcelona, Spain; 6 Departament de Biomedicina, Facultat de Medicina, Universitat de Barcelona, 08036 Barcelona, Spain; 7 Unidad Asociada (IMIM), IIBB-CSIC, 08036 Barcelona, Spain; 8 CIBERCV, ISCIII, 28029 Madrid, Spain; 9 Centro de Investigación Biomédica en Red en Enfermedades Neurodegenerativas (CIBERNED), ISCIII, 28029 Madrid, Spain

**Keywords:** liver cancer, tumor microenvironment, NLRP3, ASC, IL-1β, pyroptosis

## Abstract

**Simple Summary:**

In the past decade, tyrosine kinase inhibitors sorafenib and regorafenib have been standard treatments for advanced liver cancer. Previous studies have associated sorafenib with inflammasome activation, but its therapeutic role in patients has not been explored. Our study found that both treatments altered inflammasome-related gene expression, showing a similar transcriptomic pattern in a mouse model of liver cancer. In accordance with public databases, inflammasome genes are associated with a poorer prognosis in male liver cancer patients, without changes in females. Thus, we analyzed biopsies from hepatocellular carcinoma patients treated with sorafenib/regorafenib and confirmed inflammasome activation. In experimental models, inflammasome inhibition enhanced sorafenib effectiveness, demonstrating its potential to overcome therapy resistance and improve its efficacy for liver cancer treatment.

**Abstract:**

During the last decade, tyrosine kinase inhibitors (TKIs) sorafenib and regorafenib have been standard systemic treatments for advanced hepatocellular carcinoma (HCC). Previous data associated sorafenib with inflammasome activation. However, the role of the inflammasome in sorafenib and regorafenib signaling has not been described in liver cancer patients. For this purpose, we analyzed inflammasome-related transcriptomic changes in a murine HCC model. Our data confirmed inflammasome activation after both TKI treatments, sharing a similar pattern of increased gene expression. According to human database results, transcriptional increase of inflammasome genes is associated with poorer prognosis for male liver cancer patients, suggesting a sex-dependent role for inflammasome activation in HCC therapy. In biopsies of HCC and its surrounding tissue, we detected durable increases in the inflammasome activation pattern after sorafenib or regorafenib treatment in male patients. Further supporting its involvement in sorafenib action, inflammasome inhibition (MCC950) enhanced sorafenib anticancer activity in experimental HCC models, while no direct in vitro effect was observed in HCC cell lines. Moreover, activated human THP-1 macrophages released IL-1β after sorafenib administration, while 3D Hep3B spheres displayed increased tumor growth after IL-1β addition, pointing to the liver microenvironment as a key player in inflammasome action. In summary, our results unveil the inflammasome pathway as an actionable target in sorafenib or regorafenib therapy and associate an inflammasome signature in HCC and surrounding tissue with TKI administration. Therefore, targeting inflammasome activation, principally in male patients, could help to overcome sorafenib or regorafenib resistance and enhance the efficacy of TKI treatments in HCC.

## 1. Introduction

Despite the improvement for liver cancer patients that immunotherapy is offering, hepatocellular carcinoma (HCC) is still a primary tumor with a dismal prognosis [1,2]. In the field of systemic therapies for HCC, tyrosine kinase inhibitors (TKIs) have been the first treatment with proven benefit, while immunotherapy-based regimens recently landed as the first-line option [2]. Nevertheless, in order to further advance in therapy efficacy, not only immune-based therapies must be contemplated, but also those using TKIs should be enhanced. Recent data indicates that around 25% of HCC patients suffer treatment interruption after first-line immunotherapy regimens (Fortuny et al., poster #022 at AEEH 2024 meeting). Therefore, it is still necessary to gain better knowledge of the mechanisms involved in resistance to TKI treatments and to identify cellular markers of therapy efficacy. Importantly, TKIs such as sorafenib and regorafenib remain an appropriate treatment alternative for patients with advanced hepatocellular carcinoma (HCC) in whom atezolizumab plus bevacizumab therapy or tremelimumab–durvalumab are contraindicated or failed. Regarding these mechanisms, we have previously observed marked induction of some inflammasome-related proteins after sorafenib exposure in HCC mouse models [3,4]. Interestingly, the appearance of sorafenib-related dermatologic side effects has been linked with increased therapy success against HCC [5]. In particular, sorafenib-induced early dermatologic adverse events (DAEs) predict better outcomes in HCC patients, suggesting a role for the immune/inflammatory system in the response to TKI therapy [6,7,8].

The inflammasome activation plays a role in the development and progression of several diseases through its involvement in chronic inflammation, immune responses, and cell death [9,10]. Inflammasome activation has been implicated in liver cancer, particularly in the context of chronic liver inflammation and associated liver diseases, such as hepatitis B and C infections, alcoholic liver disease, and metabolic dysfunction-associated steatohepatitis (MASH) [11]. Inflammasome activation in liver cells, including hepatocytes, hepatic stellate cells, and immune cells, can lead to the release of pro-inflammatory cytokines, such as IL-1β and IL-18 [12,13,14,15], which can promote inflammation and contribute to cancer promotion in the tumor microenvironment [16]. Activation of specific inflammasome complexes, such as the NLRP3 inflammasome, has been observed in liver cancer and has been associated with tumor growth, angiogenesis, and metastasis [17,18], but its role has not been analyzed in patients under HCC treatments. In contrast, cell death mediated by the NLRP3 inflammasome (pyroptosis) and increased efficiency of CD8^+^ T cells could support an inhibitory role in HCC, such as promoting Natural Killer cell tumoricidal activity [19,20,21]. Therefore, the benefits of inflammasome targeting in HCC may depend on the specific weight of these opposed mechanisms and how they are affected by a particular therapy [17,22,23].

In this sense, despite sorafenib being reported as an inflammasome inducer in cellular models of cancer [21], inflammasome intervention has not been explored in detail using HCC models, and its potential relevance in HCC patients is not sufficiently studied. Since the exact mechanisms underlying sorafenib side effects are not fully understood, it is plausible that inflammasome activation may also contribute to some of the observed dermatological manifestations. In particular, the association of early DAEs with sorafenib/regorafenib efficacy led us to tackle this issue. For this purpose, we decided to study in a murine model of HCC if transcriptomic changes in inflammasome-related genes are detectable after TKI treatment with sorafenib and regorafenib. Similar patterns of inflammasome activation were generated by both compounds, which led us to analyze tumor and adjacent liver samples for evidence of inflammasome changes in HCC patients treated with sorafenib or regorafenib. We confirmed inflammasome activation in most TKI-treated individuals, both in the HCC and the surrounding tissue. After evaluating that inflammasome inhibition could be effective in experimental HCC models to potentiate sorafenib anticancer activity, our results point to the potential use of inflammasome-directed therapy as a strategy to increase sorafenib or regorafenib efficacy in HCC treatment.

## 2. Materials and Methods

### 2.1. Reagents

Dulbecco’s modified Eagle’s medium (DMEM), Roswell Park Memorial Institute 1640 medium (RPMI), trypsin–EDTA, penicillin–streptomycin, and dimethyl sulfoxide (DMSO), MTT (3-(4,5-dimethylthiazol-2-yl)-2,5-diphenyl tetrazolium bromide) (M2128), and Crystal Violet (C0755) were purchased from Sigma-Aldrich (St. Louis, MO, USA). Sorafenib (BAY 43-9006, Nexavar) and regorafenib (BAY 73-4506, Stivarga) are manufactured by Bayer (Leverkusen, Germany). MCC950 was purchased from RayBiotech (Peachtree Corners, GA, USA).

### 2.2. Tumor Animal Models

All animal procedures were performed according to protocols approved by the Animal Experimentation Ethics Committee from the University of Barcelona (ethics code: #11415). For the subcutaneous tumor model, male Swiss nude mice, 5–6 weeks old, were kept under pathogen-free conditions with free access to standard food and water. BCLC9 cells (2.5 × 10^6^) were injected subcutaneously into the flanks of mice in 100 μL DMEM, as previously reported [24]. Treatments with sorafenib (30 mg/kg body weight), regorafenib (30 mg/kg), or vehicle (12.5% Cremophor, 12.5% ethanol, 75% sterile saline) were delivered daily via oral gavage. For the immunocompetent model, 2.5 × 10^6^ Hepa1-6 cells were injected subcutaneously into the flanks of C57BL/6J. Mice were treated daily via oral gavage with sorafenib (10 mg/kg body weight) and MCC950 (20 mg/kg). Tumors were measured periodically with a Vernier caliper, and the volume was calculated as length × width^2^ × 0.5.

### 2.3. HCC Patient Study and ATLAS Database Information

Tumor and cirrhotic tissue derived from 19 patients diagnosed with HCC and treated at the Hepatic Oncology Unit (BCLC), Liver Unit of the Hospital Clinic of Barcelona. Patient data is included in Supplementary Table S1. The study was reviewed and approved by the Hospital Clinic of Barcelona Board of Clinical and Experimental Research (HCB/2013/8351 and HCB/2017/1016) and complied with the provisions of Good Clinical Practice guidelines. Patients gave informed consent according to the principles embodied in the Declaration of Helsinki.

Data showing survival probability in HCC patients depending on the level of expression of inflammasome-related genes were retrieved from the “Pathology” section of the open database Human Protein Atlas, where the impact of specific protein levels for the survival of patients with cancer is shown (https://www.proteinatlas.org (accessed on 5 September 2023)). Both sexes were analyzed separately.

### 2.4. RNA Isolation and Gene Array

Total RNA was isolated with TRIzol reagent. One µg of RNA was reverse-transcribed with iScript™ cDNA Synthesis Kit (Bio-Rad, Berkeley, CA, USA). A predesigned 384-well human inflammasome panel (SAB Target List, H384 Cat# 10034515) for SYBR Green detection (Bio-Rad) was used following the manufacturer’s instructions, as previously reported [25].

### 2.5. Immunohistochemical Staining

Tumors were fixed with 10% neutral buffered formalin for 48 h, paraffin-embedded, and cut in 5-μm sections. Heat-induced antigen retrieval was performed in citrate buffer, and after blocking endogenous peroxidase, slides were incubated with primary antibody anti-PCNA antibody (PC10) (1:200 dilution, sc-56, Santa Cruz Biotechnology) as previously indicated [26]. Then, slides were incubated with a biotinylated antibody and developed with the ABC-HRP Kit (Vector Laboratories, Burlingame, CA, USA) and peroxidase substrate DAB (Sigma-Aldrich, St. Louis, MO, USA). Afterward, slices were examined with a Zeiss Axioplan microscope equipped with a Nikon DXM1200F digital camera. The PCNA cell count was quantified in four randomly selected fields from each animal and analyzed using ImageJ 1.54e software.

### 2.6. Cell Culture and Biochemical Analysis

Human hepatoma HepG2, Hep3B, and murine hepatoma cell line Hepa1-6 (European Collection of Animal Cell Cultures (ECACC)) were grown in Dulbecco’s modified Eagle’s medium (DMEM) with 10% FBS and penicillin–streptomycin (100 U/mL–100μg/mL) at 37 °C and 5% CO_2_. Human BCLC9 cell lines [27] were cultured in DMEM and Nutrient Mixture F-12 HAM (Sigma-Aldrich, St. Louis, MO, USA) supplemented with 10% FBS, 1% essential amino acids, 2 mm l-glutamine, 50 units/mL penicillin, and 50 μg/mL streptomycin and 1 mm pyruvic acid. THP-1 monocytes were differentiated into macrophages with 200 nM phorbol 12-myristate 13-acetate (PMA, Sigma-Aldrich) during 72 h incubation followed by 24 h incubation in RPMI medium (2.5% FBS).

For the MTT Assay: Cell viability was determined by MTT (3-(4,5-dimethylthiazol-2-yl)-2,5-diphenyl tetrazolium bromide) assay. In total, 1 × 10^4^ cells/well were seeded in a 96-well plate and incubated at 37 °C and 5% CO_2_. Cells were treated with sorafenib and MCC950 for 16 h, and MTT reagent was added (10 µL 5 mg/mL) and incubated for 2 h. Dried formazan crystals were dissolved with 100 µL of 1-propanol, and absorbance was measured at 570 and 630 nm in a plate reader (Multiskan^®^ Spectrum, Thermo Fisher Scientific, Rockford, IL, USA).

### 2.7. Cytokine Array and ELISA

Mouse Cytokine Array (GSM-CYT-1, Raybiotech) was used for the measurement of 20 mouse cytokines. Serum samples from mice were hybridized according to manufacturer instructions and analyzed using an Innoscan 710 laser scanner (Innopsys, Carbonne, France) for glass slides and Mapix 9.0.0 software used for quantifications. IL-1β levels were determined in medium by specific sandwich ELISA using commercial kits (#DY401 DuoSet ELISA; R&D Systems) and following the manufacturer’s instructions.

### 2.8. Three-Dimensional Tumor Liver Spheroid Generation

Hep3B cellular spheroids were generated and plated in 96-well agarose-coated plates. Tumor spheroids were kept at 37 °C and 5% CO_2_ for seven days, and spheroid growth was monitored [4,28]. Hep3B tumoroids were treated in regular culture medium with sorafenib (1 µM) and the inflammasome inhibitor MCC950 (1 µM) for seven days or in conditioned medium from sorafenib-treated THP-1 cells (10 µM, 24 h), concentrated using Protein Concentrators 3 K (Thermo Scientific, Waltham, MA, USA, Ref#88515), and co-incubated with the recombinant IL-1 receptor antagonist Anakinra (0.5 µM) from MedChem (Cat# HY-108841).

### 2.9. Statistical Analyses

Statistical comparisons were usually performed using unpaired 2-tailed Student’s *t*-test and 1-way ANOVA followed by Newman–Keuls multiple comparison test (GraphPad Prism). A *p*-value less than 0.05 was considered significant. Results are expressed as means ± standard deviation and *n* = 3 unless indicated.

## 3. Results

### 3.1. Sorafenib and Regorafenib Induction of Inflammasome-Related Genes Is Similar, Exhibiting Analogous Transcriptomic Signature

Resistance to TKI treatments compromises the efficiency of cancer therapy. The knowledge of the mechanisms involved in acquired drug resistance could greatly improve treatment efficacy. We have previously observed marked induction of specific inflammasome-related proteins after sorafenib exposure in HCC mouse models [3]. As activation of inflammasomes has been related to inflammatory-based side effects in cancer treatment [29,30,31], we hypothesize that this pathway could be implicated in sorafenib-related dermatologic side effects, a prognostic marker of therapy success against HCC [5,6].

**Figure 1:**
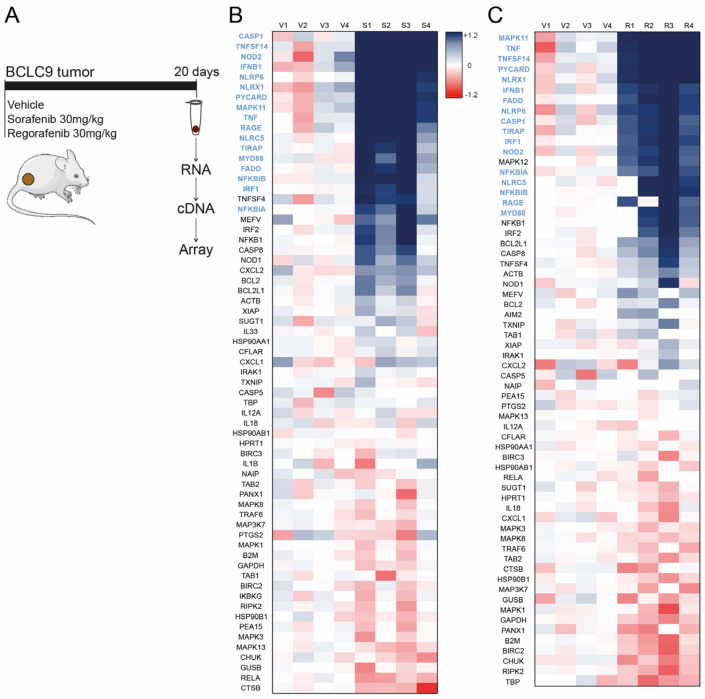
Sorafenib- and regorafenib-induced upregulation of inflammasome-related genes in a murine HCC model. (**A**), Mice bearing BCLC9 tumors were treated with vehicle, sorafenib, or regorafenib for 20 days, and cDNA was generated after sacrifice. (**B**,**C**), Heat map representation of the transcriptomic analysis of genes related to inflammasome activation in BCLC9 tumors from nude mice (n = 4) treated for three weeks with vehicle (V1–4) and sorafenib (S1–4) (**B**), or vehicle (V1–4) and regorafenib (R1–4) (**C**).

To assess this possible connection, we evaluated the impact of the inflammasome activation on the resistance to the first- and second-line systemic treatment of HCC. To do so, we analyzed the transcriptomic changes in inflammasome-related genes in the mRNA from mice xenografts after sorafenib treatment of BCLC9 tumors (Figure 1A) and compared these values with the numbers obtained after regorafenib treatment, another TKI used in second-line HCC therapy, that also causes dermatologic side effects.

Interestingly, when Swiss nude mice bearing subcutaneous BCLC9 tumors were treated with sorafenib (Figure 1B), a substantial increase in several inflammasome-related genes was detected in the transcriptomic analysis of the tumors using a qPCR array (Bio-Rad, Cat#10034515). More importantly, a similar pattern of increase was observed in BCLC9 tumors obtained from mice treated with regorafenib (Figure 1C), indicating that inflammasome stimulation is a common mechanism in the activity of both TKIs.

Of note, while the genes downregulated by sorafenib and regorafenib were different for both compounds, a similar upregulation of inflammasome-related genes, such as CASP1, PYCARD (ASC), TNFSF14 (Light), or nucleotide-binding domain and leucine-rich repeat-containing genes (NLRs) was detected.

To better illustrate this similitude, the 15 genes showing higher upregulation after sorafenib or regorafenib treatment are presented in blue in Figure 1. This remarkable coincidence in gene induction for both TKIs may suggest that a transcriptomic signature could also be detectable after therapy with sorafenib and regorafenib in HCC patients.

### 3.2. Transcriptional Increase of Inflammasome-Related Genes Is Associated with Poorer Prognosis Only for Male Liver Cancer Patients, According to Database Results

**Figure 2:**
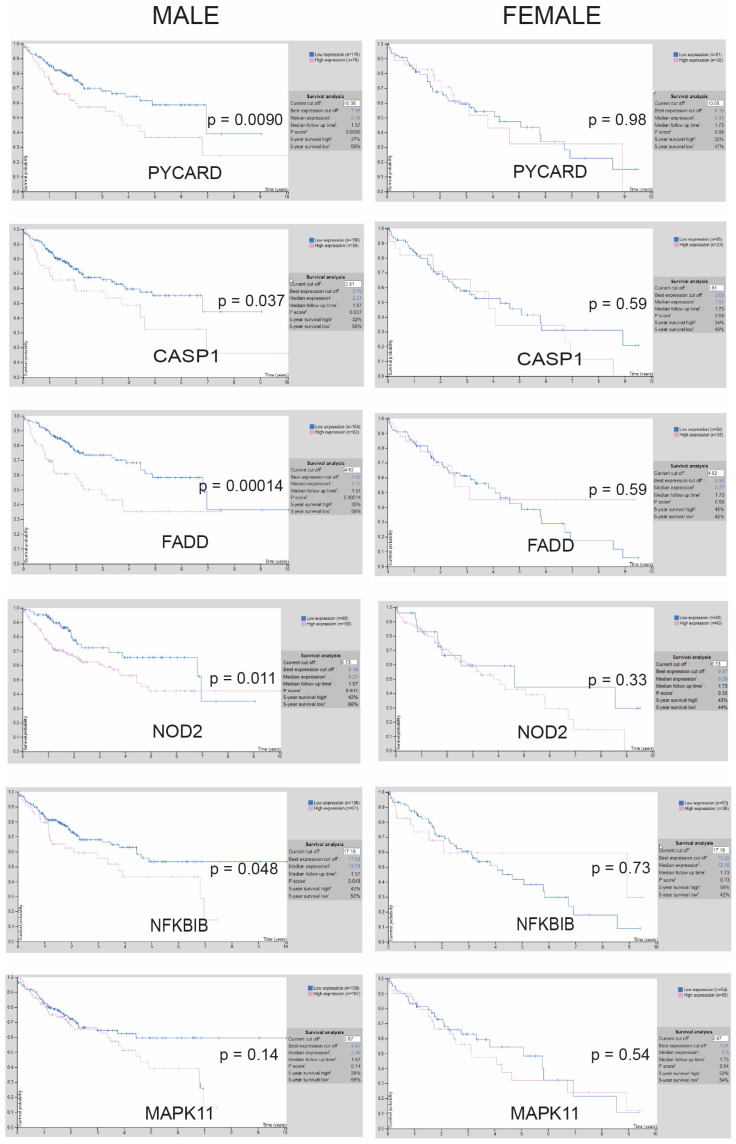
Representation of survival probability depending on gene expression of inflammasome-related genes in male and female HCC patients. Images for individual gene expression (blue, high; purple, low) in male (**left**) and female (**right**) patients with HCC cancer were obtained from the open database Human Protein Atlas, and statistical significance (*p*) was indicated for each group.

To search for data supporting the importance of this pathway in HCC patients, we examined the relationship between key inflammasome-related genes and survival from liver cancer in the Human Atlas database. We observed that most of the genes more transcriptionally altered by sorafenib administration, according to the results we obtained in sorafenib-treated tumors, such as PYCARD, CASP1, NOD2, FADD, or NFKBIB were associated with unfavorable outcomes for HCC patients (Figure 2 and enhanced images available in Supplemental Figures S1 and S2).

Interestingly, a pronounced gender bias was observed. The association of high inflammasome gene expression with poor liver cancer prognosis was restricted to male patients suffering from liver cancer since changes in the expression of these genes did not seem to be reflected in the survival of female patients.

This observation may suggest a sex-dependent role for inflammasome activation in HCC therapy, as previously reported for other inflammasome-related pathologies [32,33], and should probably be considered for the design of inflammasome-based therapies applied to HCC patients. Since most of our patients were male, this prospect facilitated focusing our analysis principally on male patients in order to check for inflammasome activation after sorafenib or regorafenib treatment.

### 3.3. Sorafenib and Regorafenib-Treated HCC Patients Exhibit Transcriptomic Induction of the Inflammasome Pathway

**Figure 3:**
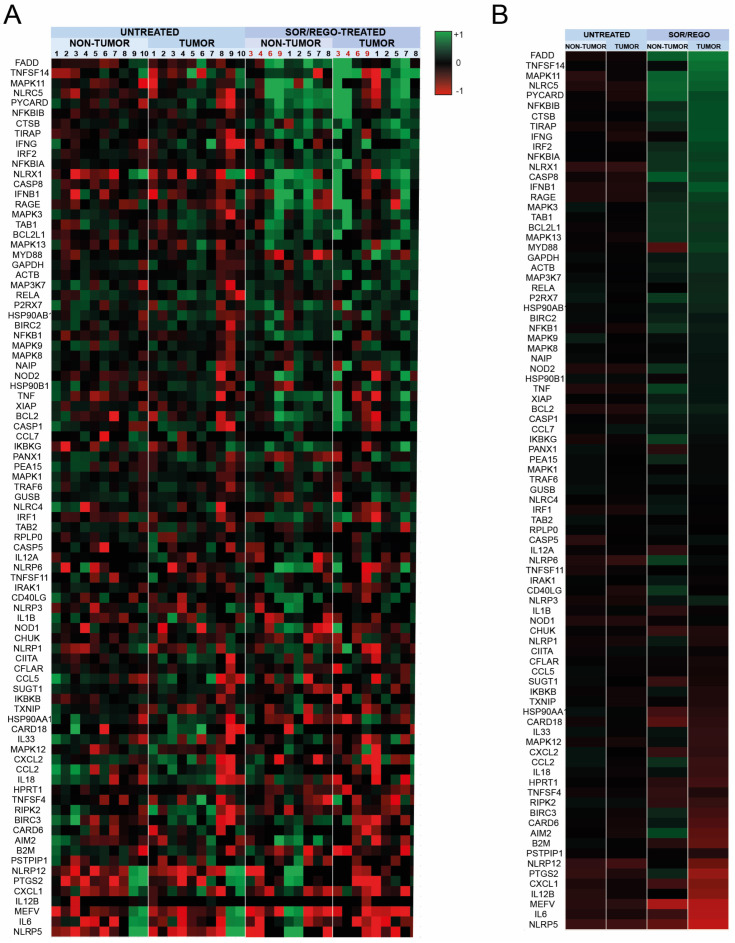
Transcriptomic changes in inflammasome-related genes in sorafenib/regorafenib-treated HCC patients. cDNAs obtained from HCC tumor and adjacent tissue biopsied from patients under sorafenib/regorafenib treatment or untreated were analyzed using a microarray for mRNA expression of inflammasome-related genes. In (**A**), heat map representation of individual samples from untreated patients (*n* = 10) or after sorafenib/regorafenib administration (*n* = 9). In (**B**), representation of the average expression of each group.

To validate this assumption, we analyzed the mRNA in tumor and adjacent liver tissue from biopsied HCC patients before (*n* = 10) systemic treatment and after (*n* = 9) receiving sorafenib as first-line (*n* = 4) or regorafenib as second-line after sorafenib treatment (*n* = 5). As commented above, the transcriptomic changes in inflammasome-related genes were quantified using PrimePCR arrays, while heat map figures expressed in logarithmic values were used to better visualize the results (Figure 3A).

As observed previously in the xenograft experiments, while no differences were observed in liver tissue or tumor samples from untreated HCC patients, a pattern of inflammasome activation is exhibited in both tissues (Figure 3B) treated with sorafenib (samples #3, #4, #6, #9) and with regorafenib after sorafenib (samples #1, #2, #5, #7, #8).

As previously shown in mouse models, both sorafenib and regorafenib trigger similar inflammasome responses, as denoted by the similar pattern of activation observed in each group. Interestingly, both sorafenib and regorafenib-induced inflammasome activation affect the tumoral and the non-tumoral area since inflammasome-related genes are upregulated in both liver samples after sorafenib/regorafenib administration (Figure 3B). This result suggests that not only the tumor but also the whole liver, which in HCC patients frequently deteriorates up to a cirrhotic stage, is sensing sorafenib and regorafenib treatment.

Of note, regarding a potential relationship between DAE occurrence in patients and detection of the inflammasome activation in liver or HCC samples, no significant connection was detected (Supplemental Figure S3). The presence of an inflammasome transcriptional pattern exhibited by patients without DAE (*n* = 7) was similar to the signature observed in patients suffering DAEs (*n* = 3, 2 males and 1 female). The lack of presence of DAEs in patients with high inflammasome activation ratios, particularly in TKI-treated tumors, does not support these as related events. Although our results do not link an increased inflammasome signature to DAE appearance in sorafenib or regorafenib-treated HCC patients, we do not discard its use as an early biomarker of TKI efficacy since an inflammasome-related pattern is clearly associated with sorafenib or regorafenib therapy.

Therefore, we decided to investigate whether inflammasome activation could be a mechanism triggered by sorafenib and regorafenib that participates in their antitumoral action.

### 3.4. Inflammasome Inhibition Potentiates Sorafenib Efficacy in a Murine Subcutaneous Model of Human HCC

Inflammasome modulators, in particular specific inhibitors such as MCC950, are interesting compounds for the pharmaceutical industry as they may be useful against autoinflammatory and autoimmune diseases, including neurodegenerative diseases and metabolic disorders, or cancer treatment [34]. Since our results indicate that inflammasome activation is induced by sorafenib and regorafenib during HCC therapy, we decided to evaluate the inflammasome contribution in an experimental model of HCC using the patient-derived cell line BCLC9.

**Figure 4:**
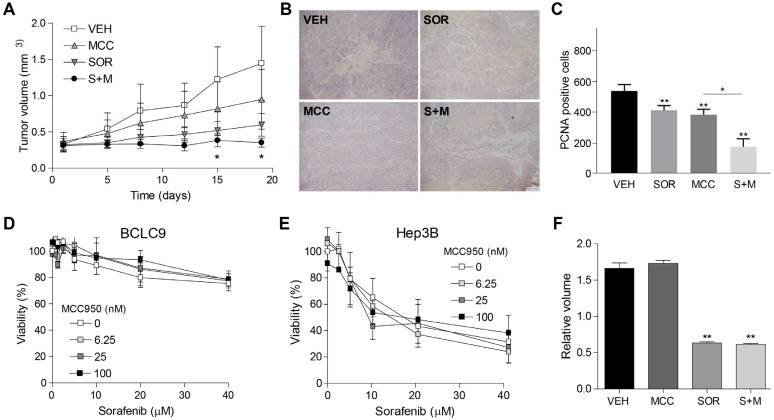
Inflammasome inhibition increased sorafenib efficacy in a BCLC9 HCC murine model. (**A**), Swiss nude mice bearing BCLC9 tumors were treated with vehicle (VEH, *n* = 4), inflammasome inhibitor MCC950 (MCC, *n* = 4), sorafenib (SOR, *n* = 6), or both (S + M, *n* = 6) and changes in tumor volume was measured. (**B**,**C**), Representative images of PCNA expression in treated BCLC9 tumors were taken and quantified using ImageJ software. * *p* < 0.05; ** *p* < 0.01 vs. vehicle-treated mice. (**D**,**E**), Effect of MCC950 on sorafenib-treated BCLC9 and Hep3B cells after 24 h. (**F**), Hep3B spheroids were grown and treated for 7 days with sorafenib (1 µM) and MCC950 (1 µM). ** *p* < 0.01 vs. vehicle.

For this purpose, mice bearing the BCLC9 subcutaneous tumors were treated with MCC950 alone or in combination with sorafenib. Our results indicated that inflammasome inhibition by MCC950 potentiates sorafenib antitumoral activity against HCC (Figure 4A). Necrotic areas are particularly present in sorafenib + MCC950 treated tumors (Figure 4B). While the number of PCNA-positive cells significantly diminished after sorafenib or inflammasome inhibitor treatments, they were further decreased in sorafenib + MCC950 treated mice (Figure 4C). Of note, MCC950 caused no hepatic damage, as denoted by unchanged transaminase levels in serum and observed after H&E staining of liver biopsies. These results suggest that inflammasome activation could be a mechanism induced by sorafenib and regorafenib treatment that reduces the efficacy of both TKIs, pointing to inflammasome inhibition as a potential strategy to block drug resistance and improve drug effectiveness, potentiating the antitumor effect of sorafenib and regorafenib against HCC. To test if the reduction in tumor growth induced by inflammasome inhibition was due to a direct effect on liver tumor cells, we evaluated the influence of inflammasome inhibition on the cytotoxicity induced by sorafenib.

No changes in the cytotoxic effect of sorafenib were observed after MCC950 addition in BCLC9 cells (Figure 4D). Moreover, a similar lack of inflammasome-dependent effect was found in other human hepatoma cell lines, such as Hep3B (Figure 4E), treated with sorafenib. To better test the direct effect of the inflammasome inhibition in the tumoral growth of HCC cells, Hep3B spheroids were made and treated with sorafenib for seven days. No effect of MCC950 was observed on tumoroid development or after sorafenib administration (Figure 4F).

Therefore, in vivo tumor reduction seems to be associated with inflammasome inhibition in non-tumoral cells since MCC950 administration does not affect in vitro sorafenib-induced cell death in liver tumor cells growing alone.

### 3.5. Inflammasome Inhibition on Tumor Microenvironment Increases Sorafenib Efficacy in a Syngeneic Mouse Model

**Figure 5:**
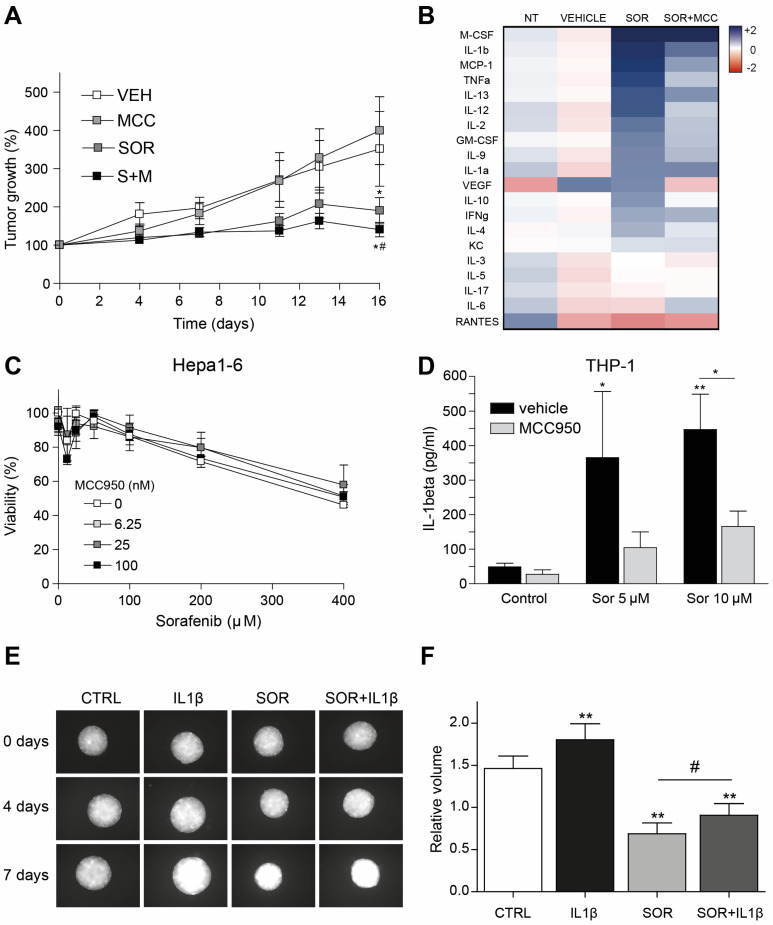
IL1β, inflammasome-dependently produced by activated macrophages, promotes tumoroid growth in human HCC cells. (**A**), Immunocompetent mice bearing Hepa1-6 tumors were treated with vehicle (VEH, *n* = 4), inflammasome inhibitor MCC950 (MCC, *n* = 4), sorafenib (SOR, *n* = 6) or both (S + M, *n* = 6) and changes in tumor volume was measured. * *p* < 0.05 vs. vehicle-treated mice. # *p* < 0.05 vs. sorafenib-treated mice. (**B**), Mouse Cytokine Array was hybridized with serum from treated mice and quantified using Mapix software (*n* = 4). (**C**), Effect of inflammasome inhibition on sorafenib-treated Hepa1-6 cells after 24 h (*n* = 3). (**D**), IL1β production by activated THP-1 macrophages after sorafenib treatment and inflammasome inhibition (*n* = 3). * *p* < 0.05; ** *p* < 0.01 (**E**,**F**), Representative images of Hep3B tumoroids treated with sorafenib (1 µM) and IL-1β (0.25 µg/mL) for 7 days were taken and quantified using ImageJ software. * *p* < 0.05; ** *p* < 0.01 vs. CTRL tumoroids. # *p* < 0.05 vs. sorafenib-treated tumoroids.

To verify the efficacy of inflammasome inhibition to potentiate the effect of sorafenib in HCC treatment in an immunocompetent model where tumor microenvironment could play a more relevant role, we used a syngeneic mouse model with a murine hepatoma-derived tumor. To do so, C57BL/6 mice were subcutaneously injected with Hepa1-6 cells and treated with sorafenib and the inflammasome inhibitor MCC950. Once again, the efficacy of sorafenib was potentiated by inflammasome inhibition, decreasing Hepa1-6 tumor growth in C57BL/6 mice (Figure 5A). Interestingly, analysis of inflammatory cytokines in the serum of these mice revealed an important increase after sorafenib administration that was diminished by MCC950 administration (Figure 5B).

Besides a marked reduction in IL-1β production after inflammasome inhibition, strong decreases in TNF and MCP-1, among other inflammatory proteins, were detected, suggesting an MCC950 effect on mouse macrophages.

Supporting a predominant effect of inflammasome inhibition on non-tumoral cells, as previously observed in human liver cancer cells, in vitro pre-treatment of Hepa1-6 cells with MCC950 did not alter the cytotoxic action of sorafenib (Figure 5C).

In order to test if sorafenib could act through the activation of the inflammasome in tumor-associated macrophages (TAMs), PMA-activated THP-1 cells were treated with sorafenib and MCC950, and IL-1β production was detected in the cellular media after 16 h. Sorafenib-induced IL-1β released from activated macrophages was clearly reduced by inflammasome inhibition with MCC950 (Figure 5D).

As in vitro 2D cell culture often lacks many features of cancer development, to test if the IL-1β increase could impact the tumor growth, such as hypoxia, altered metabolism, or cell contact, we cultured 3D spheroids to better mimic the in vivo context. Thus, tumor spheroids were generated using Hep3B cells (10^4^ cells), incubated in the presence of sorafenib (1 μM), and the effect of IL-1β (0.25 μg/mL) was analyzed.

Interestingly, IL-1β promoted Hep3B spheroid growth and reduced sorafenib antitumoral activity when co-administered (Figure 5E,F). These results indicate that inflammasome-induced release of IL-1β by TAMs may contribute to tumor growth of HCC cells, a mechanism that would be reduced by treatment with an inflammasome inhibitor such as MCC950 (Figure 5B).

In fact, Hep3B spheroids treated with conditioned medium from THP-1 activated macrophages, incubated with sorafenib displayed reduced tumor growth, which was further reduced either by MCC950 addition or after administration in the conditioned medium of an IL-1 antagonist such as Anakinra (Supplemental Figure S4). These results point to the targeting of the IL-1β release as a potential strategy for combination with TKI therapy, although additional research should be performed to corroborate this, particularly in the context of HCC treatment.

## 4. Discussion

In the present investigation, we show that sorafenib and regorafenib induce a comparable transcriptomic signature in inflammasome-related genes, suggesting a potential commonality in their mechanism of action. In fact, the identification of a gene induction pattern hints at the existence of a detectable transcriptomic signature following therapy with both sorafenib and regorafenib. Once observed in our cohort of patients, it would be of interest to analyze a second cohort with more individuals included. In particular, our analysis of the Human Atlas database reveals that increased transcriptional levels of inflammasome-related genes, altered by sorafenib treatment, are associated with an unfavorable prognosis in HCC patients, specifically in men. This observation suggests a potential sex-dependent role for inflammasome activation in HCC therapy, in line with reports pointing to an estrogenic control of inflammasome activity [35]. Therefore, gender-specific considerations in inflammasome-based therapeutic strategies are required when searching for a specific inflammasome signature and its association with treatment efficacy.

Regarding our cohort of patients, the evaluation of HCC samples before and after systemic treatment with sorafenib or regorafenib demonstrates a consistent pattern of inflammasome activation in both tumor and adjacent liver tissues. The activation of the inflammasome pathway in the whole liver, which is frequently deteriorated in HCC patients, indicates the inflammasome-induced response to sorafenib and regorafenib treatment from non-tumoral cells may be critical in their antitumoral action.

Inflammasome activation is associated with different pathologies and is described to participate in the action of several chemotherapeutic agents [36,37,38]. To verify if this role in sorafenib or regorafenib action is to cause cell death or a consequence of the cellular damage induced, we tested in mouse models whether inflammasome blockage could potentiate or attenuate HCC tumor growth. Our results indicated that the inhibition of the inflammasome by MCC950 enhanced the antitumoral activity of sorafenib in both subcutaneous and syngeneic mouse models of HCC. Moreover, the combination of sorafenib and MCC950 results in increased necrotic areas in tumors, reduced PCNA-positive cells, and no apparent hepatic damage, suggesting a potentiation of the antitumor effect without added toxicity. One should consider that in these models, there is no previous liver deterioration as observed in HCC patients, thus not exactly recapitulating the real-life effect of inflammasome inhibition in humans. However, inflammasome inhibition has been protective in mice models with substantial liver damage, such as in diet-induced steatohepatitis [39], so fortunately, no serious side effects should be expected.

Underscoring its relevance in the context of the tumor microenvironment, it is important that inflammasome inhibition demonstrates efficacy in an immunocompetent mouse model. In fact, the reduction of inflammatory cytokines, particularly IL-1β, following inflammasome inhibition may suggest a potential modulation of TAM activity as confirmed in activated THP-1 macrophages, supporting the notion that inflammasome inhibition acts on non-tumor cells to enhance sorafenib efficacy. Likewise, our in vitro studies on human hepatoma cell lines and 3D spheroid cultures indicate that IL-1β released by activated macrophages may contribute to tumor growth and impact the antitumoral activity of sorafenib. Although Kupffer cells (KCs) might account for a small proportion, TAMs arise primarily from circulating monocytes responding to inflammatory signals from HCC cells, differentiating into TAMs and contributing to tumor progression [40].

**Figure 6:**
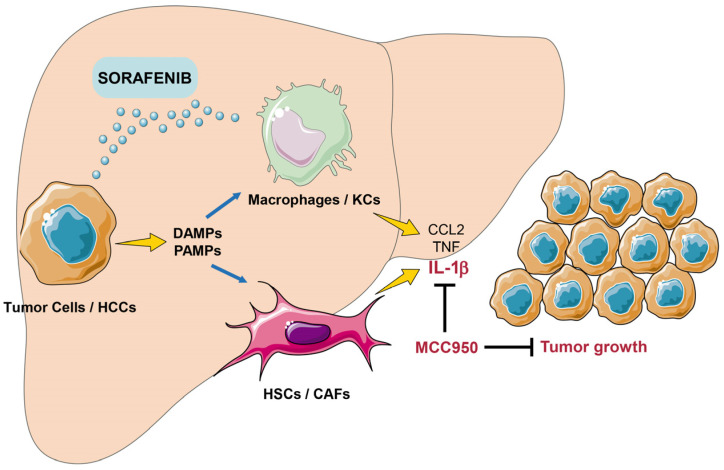
Schematic representation of inflammasome influence on HCC progression after sorafenib treatment. Sorafenib induces cell death in tumor HCC cells, frequently growing in a pro-inflammatory microenvironment. Inflammasome activation and the release of pro-tumorigenic proteins such as IL-1β reduce sorafenib efficacy in HCC therapy.

These results support an IL-1β blockade as a promising therapeutic strategy, in line with recent studies demonstrating that IL-1β secretion by macrophages facilitates HCC development [41,42]. Interestingly, a recent study identified sorafenib-induced ferroptosis as an activator of the macrophage/IL-1β/neutrophil/vasculature axis that promotes HCC progression [43]. In line with our results, depleting macrophages or neutralizing IL-1β in vivo abrogated liver cancer growth and lung metastasis, antagonizing sorafenib resistance in HCC [43]. Moreover, hepatocellular damage and pro-inflammatory liver status induce inflammasome activation in hepatic stellate cells (HSC), promoting a phenotypic switch to myofibroblasts [44], liver fibrosis and, consequently, facilitating HCC development [45] (Figure 6). Accordingly, inflammasome targeting could be an effective strategy to potentiate sorafenib efficacy in HCC therapy.

In addition to sorafenib and regorafenib, other TKIs are used for cancer therapy. For instance, lenvatinib, as well as sorafenib, has been prescribed as first-line therapy for advanced HCC [46,47]. Lenvatinib also exerts inhibition on the vascular endothelial growth factor (VEGF) pathway, its potent activity against the fibroblast growth factor (FGF) pathway a distinctive feature compared to sorafenib/regorafenib. Analogously, cabozantinib, another TKI used in HCC management, besides acting on c-MET or AXL, also inhibits VEGFR2. If this shared inhibitory effect on the VEGF pathway is involved in the promotion of inflammasome activation by other TKIs, it is an aspect that deserves to be further studied. Consequently, inflammasome- or IL-1 β-directed therapies could also be potentially considered for other cancer therapies where TKI administration has been evaluated.

## 5. Conclusions

In summary, when designing targeted therapeutic interventions for HCC, our findings underscore the importance of considering sex-dependent responses and the participation of the tumor microenvironment as relevant players. The results of the present study suggest that inflammasome activation is a frequent response to TKI treatment in HCC, such as sorafenib and regorafenib, which reduces TKI efficacy. Inflammasome inhibition holds promise as a strategy to improve sorafenib/regorafenib effectiveness and probably also for other TKIs.

## Data Availability

The data presented in this study are available in this article and Supplementary Materials.

## Supplementary Materials

The following supporting information can be downloaded at: https://www.mdpi.com/article/10.3390/cancers16081491/s1, Figure S1: Enhanced images representing the survival probability of HCC patients depending on gene expression of inflammasome-related genes (PYCARD, CASP1 and FADD); Figure S2: Enhanced images representing the survival probability of HCC patients depending on gene expression of inflammasome-related genes (NOD2, NFKBIB and MAPK11); Figure S3: DAE occurrence is not positively related to increased transcriptomic changes in inflammasome-related genes in liver or HCC samples of sorafenib/regorafenib-treated patients. cDNAs obtained from HCC tumor and adjacent tissue biopsied from patients untreated or under sorafenib/regorafenib were analyzed using a microarray for mRNA expression of inflammasome-related genes. Heat map average representation of tumor and non-tumor samples from untreated patients (*n* = 10) or after sorafenib/regorafenib administration exhibiting DAE (*n* = 3) or not (*n* = 7); Figure S4: Growth of Hep3B tumoroids treated with THP-1 conditioned medium after sorafenib (10 µM) and MCC950 (1 µM) administration and with THP-1 conditioned medium after sorafenib (10 µM) and Anakinra (0.5 µg/mL) for 7 days. * *p* < 0.05 vs. CTRL tumoroids. # *p* < 0.05 vs. sorafenib-treated tumoroids; Table S1: Data from HCC patients (untreated, n = 10, and sorafenib/regorafenib treated, n = 10) included in this study.
