# Supplementary material for: Induction of the Inflammasome Pathway by Tyrosine Kinase Inhibitors Provides an Actionable Therapeutic Target for Hepatocellular Carcinoma
Source: Cancers (Basel). 2024 Apr 13;16(8):1491. doi: 10.3390/cancers16081491 (PMC11048610; doi:10.3390/cancers16081491)
Supplement: Supplementary file 1 [file cancers-16-01491-s001.zip › cancers-2914421-supplementary.pdf]

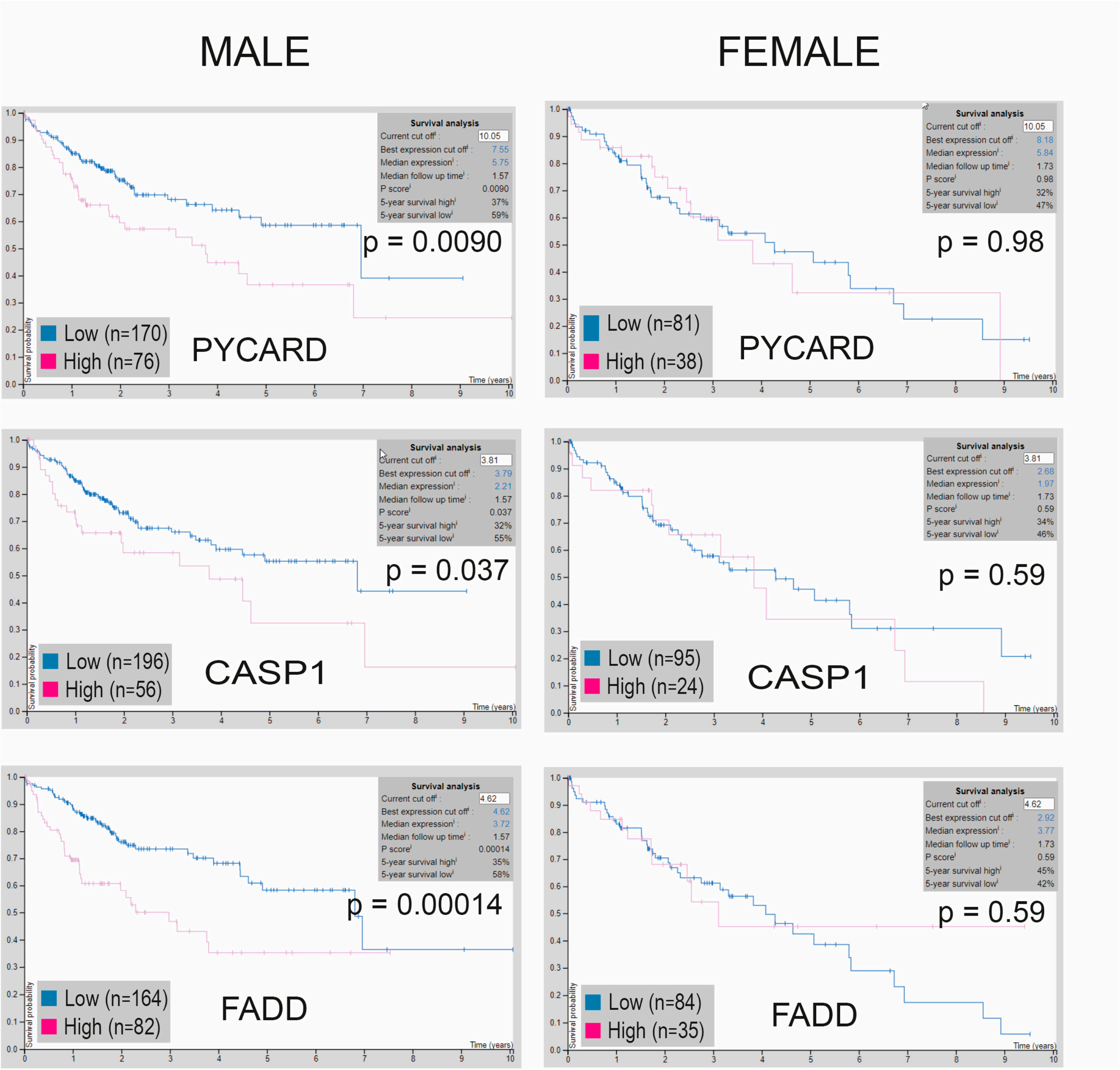

Supplemental Figure S1. Enhanced images representing the survival probability of HCC patients depending on gene expression of inflammasome-related genes.

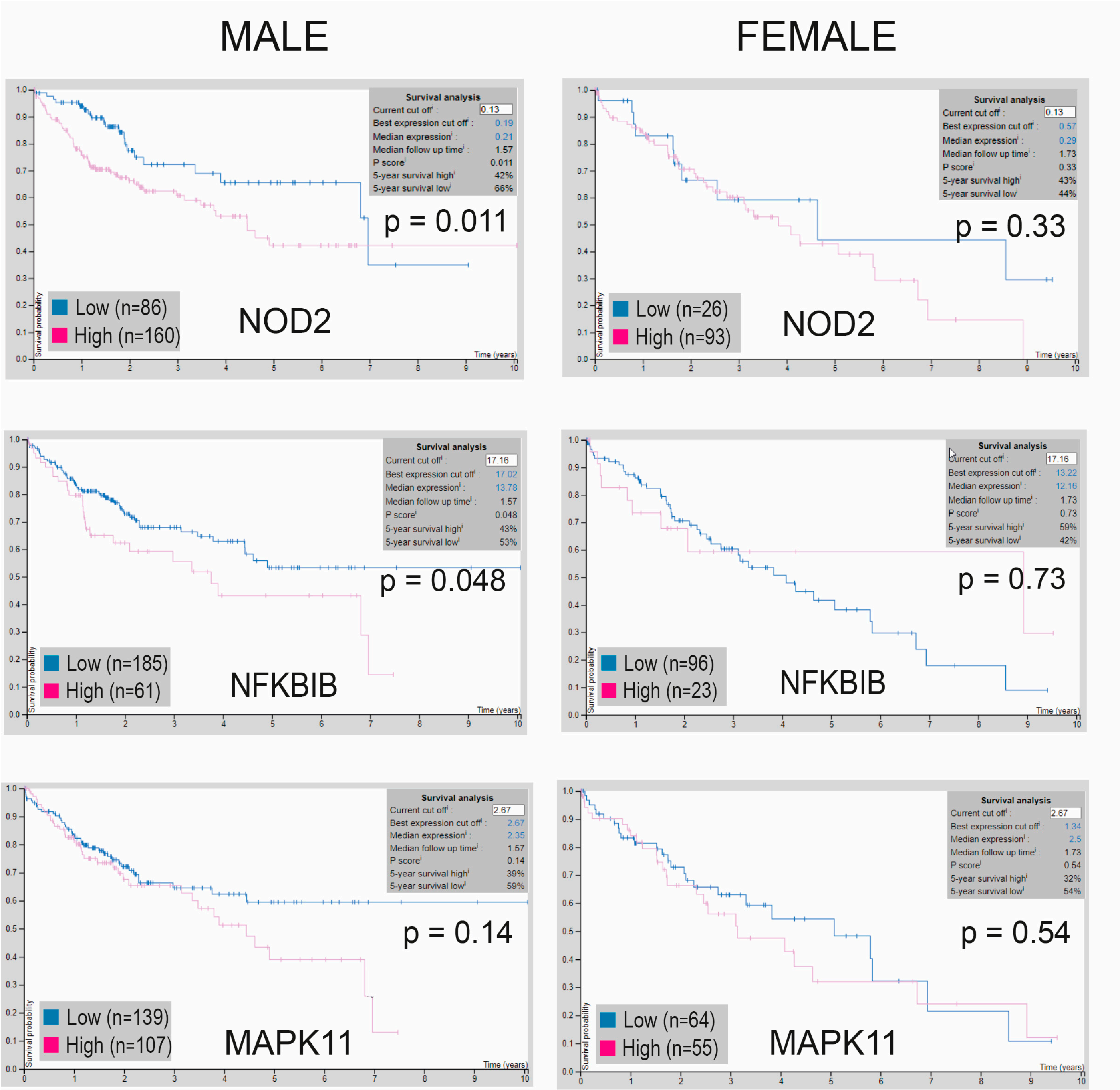

Supplemental Figure S2. Enhanced images representing the survival probability of HCC patients depending on gene expression of inflammasome-related genes.

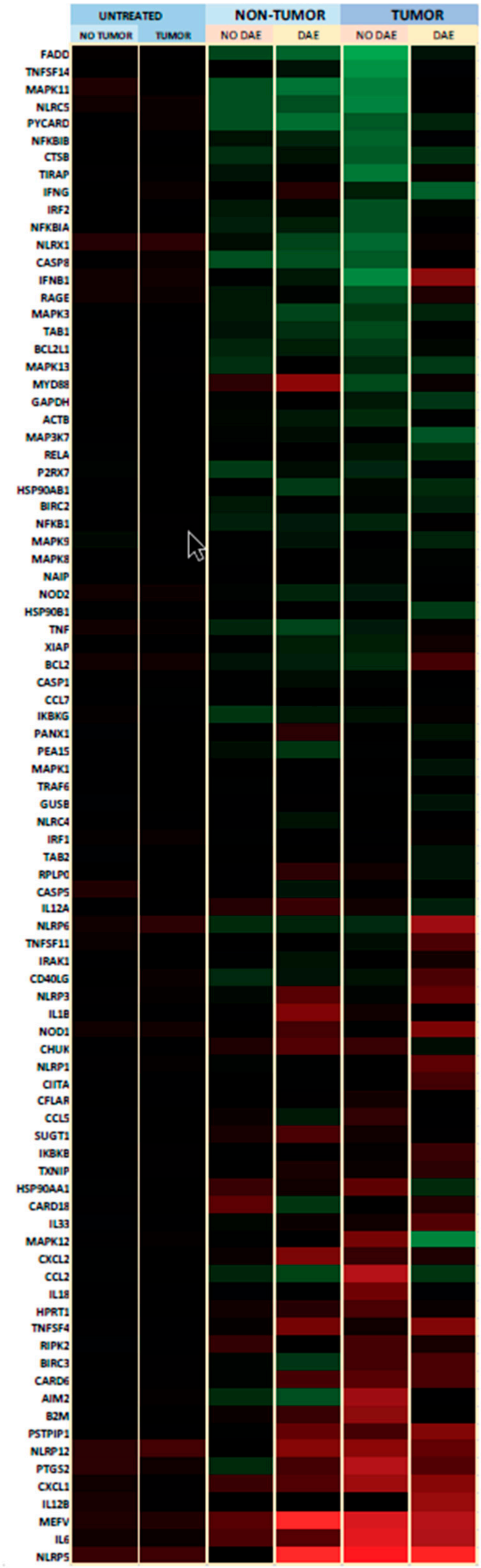

Supplemental Figure S3. DAE occurrence is not positively related to increased transcriptomic changes in inflammasome-related genes in liver or HCC samples of sorafenib/regorafenib-treated patients. cDNAs obtained from HCC tumor and adjacent tissue biopsied from patients untreated or under sorafenib/regorafenib were analyzed using a microarray for mRNA expression of inflammasome-related genes. Heat map average representation of tumor and non-tumor samples from untreated patients (n = 10) or after sorafenib/regorafenib administration exhibiting DAE (n = 3) or not (n = 7).

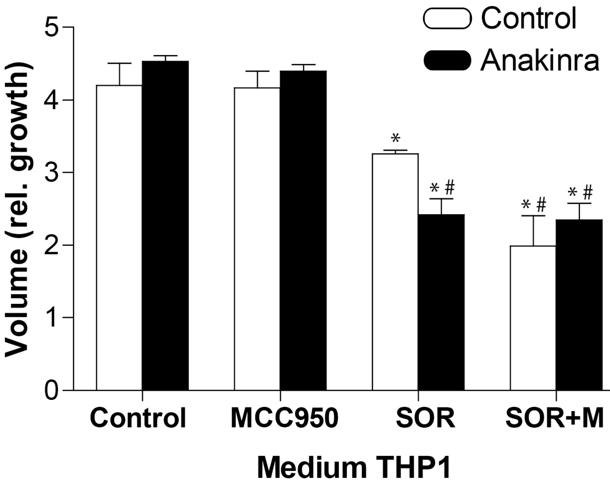

Supplemental Figure S4. Growth of Hep3B tumoroids treated with THP-1 conditioned medium after sorafenib (10 μM) and MCC950 (1 μM) administration and with THP-1 conditioned medium after sorafenib (10 μM) and Anakinra (0.5 μg/mL) for 7 days. * $p < 0.05$ vs. CTRL tumoroids. # $p < 0.05$ vs. sorafenib-treated tumoroids.

Supplemental Table S1. Data from HCC patients (untreated, n = 10, and sorafenib/regorafenib treated, n = 10) included in this study. M: male, F: female; HCV: hepatitis C virus; HBV: hepatitis B virus; MASH: metabolic dysfunction associated steatohepatitis; DAE: dermatologic adverse events; BCLC: Barcelona Clinic Liver Cancer.

Sample	Treatment	Sex	Age	Etiology	DAE	BCLC	Child-Pugh	Ascites	Survival (days)
Untreated-1	Untreated	M	54	HCV	NA	BCLCp-C2	A	NO	NA
Untreated-2	Untreated	M	62	HCV-Alcohol	NA	BCLCp-C2	A	NO	NA
Untreated-3	Untreated	M	58	HCV	NA	BCLCp-C1	A	NO	NA
Untreated-5	Untreated	M	40	HBV	NA	BCLCp-B	A	NO	NA
Untreated-6	Untreated	M	65	MASH	NA	BCLCp-C2	A	NO	NA
Untreated-7	Untreated	M	82	HCV	NA		A	NO	NA
Untreated-8	Untreated	M	69	NASH	NA	BCLCp-C1		NO	NA
Untreated-8	Untreated	M	84	HCV-Hemocromatosis	NA		A	NO	NA
Untreated-9	Untreated	M	66	MASH - Alcohol	NA	BCLCp-C2	A	NO	NA
Untreated-10	Untreated	M	71	Alcohol	NA	BCLCp-C2	A	NO	NA
Treated-1	Regorafenib	M	74	HBV	NO	BCLCp-C2	A	NO	902
Treated-2	Regorafenib	M	67	Alcohol	NO	BCLCp-C1	A	YES	1346
Treated-3	Sorafenib	M	73	NASH	NO	BCLCp-C2	A	NO	797
Treated-4	Sorafenib	M	59	HCV	NO	No PD	A	NO	3216
Treated-5	Regorafenib	M	61	HCV	NO		A	NO	400
Treated-6	Sorafenib	M	62	HBV-Alcohol	NO	BCLCp-C2	A	NO	912
Treated-7	Regorafenib	M	61	HCV-Alcohol	NO	BCLCp-B	A	NO	891
Treated-8	Sorafenib	M	56	HCV-Alcohol	YES	BCLCp-B	A	NO	670
Treated-9	Sorafenib	M	61	MASH	YES	BCLCp-B	A	NO	1103
Treated-10	Regorafenib	F	68	HCV	YES	BCLCp-C1	A	NO	612
